# Primary care physicians’ concerned voices on sickness certification after a period of reorganization. Focus group interviews in Sweden

**DOI:** 10.1080/02813432.2020.1753341

**Published:** 2020-04-21

**Authors:** Kristina Bengtsson Boström, Karin Starzmann, Anna-Lena Östberg

**Affiliations:** a Department of Public Health and Community Medicine/Primary Health Care, Institute of Medicine, Sahlgrenska Academy, University og Gothenburg, Gothenburg, Sweden;; b Regionhälsan R&D Centre Skaraborg Primary Care, Skövde, Sweden;; c Department of Behavioral and Community Dentistry, Institute of Odontology, Sahlgrenska Academy, University of Gothenburg, Gothenburg, Sweden

**Keywords:** Focus group interviews, physicians, primary health care, qualitative research, sick leave, sickness certification

## Abstract

**Objective:** This study explored the views of primary health care (PHC) physicians on sickness certification after reforms in 2005 prompted by the Swedish government to increase the quality and decrease the inequalities, and costs of sickness certification.

**Design:** Qualitative design with focus group interviews. Data were analysed using qualitative content analysis.

**Setting:** Urban and rural PHC centres in Region Västra Götaland, Sweden.

**Subjects:** GPs, interns, GP trainees and locums working in PHC centres 2015. Six focus group interviews with 28 physicians were performed.

**Main outcome measures:** Experiences and reflections about the sickness certification system.

**Results:** The latent content was formulated in a theme: ‘The physicians perceived the sickness certification process as emotive and a challenge to master with differing demands and expectations from authorities, management and patients’. Sickness certification could be easy in clear-cut situations or difficult when other factors besides the pure medical were ruling the decisions. The physicians’ coping strategies for the task included both active measures (cooperation with health care staff and social insurance officers) and passive adaptation (giving in or not caring too much) to the circumstances. Proposals for the future were to transfer lengthy sickness certifications and rehabilitation to specialized teams and increase cooperation with rehabilitation coordinators and social insurance officers.

**Conclusions:** Political decisions on laws and regulations for sickness certification impacted the primary health care making the physicians’ work difficult and burdensome. Their views and suggestions should be carefully considered in future organization of primary care.

KEY POINTS

In 2005 Swedish government introduced reforms to decrease the inequalities and costs of sickness certification and facilitate the physicians’ work.

- Focus group interviews with Swedish primary care physicians revealed that sickness certification was challenging due to differing demands from authorities, management and patients.
- Coping strategies for the sick-listing task included both active measures and passive adaptation to the circumstances.
- A proposal for future better working conditions for physicians was to transfer lengthy sickness certifications and rehabilitation to specialized teams.

## Introduction

Sickness certification has since long been considered problematic to handle by physicians in many Western countries as summarized in two reviews [1,2]. Conflicts between physicians, patients and different stakeholders in the rehabilitation process were described as dominating causes for the problems in 18 studies, both qualitative and quantitative, from the UK and Scandinavia [1]. The different roles of the stakeholders have to be clarified and the access to occupational health and rehabilitation services improved. One review [2] including 56 studies with different designs from the Nordic countries and UK showed that well-validated tools or procedures to support physicians in sickness certification were lacking especially in patients with pain and fatigue, where clinical findings were lacking. In the Netherlands the physicians are not handling sickness certifications, but instead physicians employed by social security insurance companies or occupational physicians. The findings highlighted the need for functional assessment and collaboration with physiotherapists as well as training for both current and future physicians.

The Swedish government in 2005 took action *via* economic incentives, one billion SEK (94 million €) per year to decrease the frequency of sick leave and to diminish the inequalities in assessment for sickness certification [3]. When the physician issues a sickness certificate, which in Sweden is mandatory from the eighth day of a patient’s illness, a time-scheduled rehabilitation process starts in which also employers and social insurance officers play important roles in facilitating the patients’ return to work (RTW). This rehabilitation process for sickness benefit had an absolute time limit (2.5 years), which was annulled in 2016 [4]. If the return is problematic, specifically educated rehabilitation coordinators (nurses, vocational therapists or physiotherapists) and/or multi-modality rehabilitation teams (MMR, including vocational therapists, physiotherapists, psychologists and specialized physicians) can be called upon. The rehabilitation coordinators support the physicians in the assessing of the work capacity of patients and coordinates actions from all stakeholders during rehabilitation. A national extensive education of physicians took place comprising the sickness certification process, the use of a decision support introduced by the National Board of Health and Welfare [5] and the International Classification of Functioning (ICF) from WHO [6]. These activities were introduced because issued certificates had been found to largely lack the information that officials at the Swedish Social Insurance Agency need to make decisions on sickness benefits [7]. Another reform with possible implications for sickness certification was a law from 2008 that sanctioned free choice of caregiver in primary health care (PHC), combined with a pay-for-performance system [8].

Follow-up of the effects of the Swedish reforms showed that the costs of sickness benefit declined during the first years, thereafter it has been fluctuating [9]. Studies on the sickness certification practice have shown that many certificates still are incomplete [10–12]. Swedish physicians’ views on sickness certification and rehabilitation after the reforms have been conflicting. A study using questionnaires distributed to physicians in different specialties in Sweden revealed that sickness certifications were regarded as too extensive and problematic, especially among GPs [13]. A Swedish focus group interview study showed that GPs and GP trainees were positive to include other healthcare professionals in the patient’s rehabilitation process [14]. Still, the patients are the prime sources of information about themselves as emphasized in a study where recorded dialogues between patients and physicians were assessed by GPs from different European countries [15].

Costly reforms aiming to optimize the sickness certification process need to be further evaluated. Earlier studies on sick leave reviewed above have mostly included experienced GPs from primary health care (PHC) as participants. Today, there is an array of other categories of physicians, locums and physicians during education working in Swedish PHC, often with varying levels of experience in issuing sickness certifications [16]. Their thoughts and experience of the certification process may introduce a broader perspective in this matter. Therefore, the aim of this study was to explore the views of PHC physicians of all categories on sickness certification after the reforms in Swedish social insurance in 2005.

## Material and methods

### Setting and design

The study was performed in 2015 in PHC in Västra Götaland, a region with 1.6 million inhabitants. A qualitative design was used to get an understanding of the experiences and views of the participants.

#### Participants

PHC physicians educated in Sweden or abroad, from private and public PHC centres (PHCC) in urban and rural areas were recruited by purposive sampling. They represented different professional experiences: general practitioners (GP), locum physicians, GP trainees and interns. Managers of PHCC were contacted and physicians willing to participate were further informed.

### Data collection

Data were collected by focus group interviews in order to take advantage of the interaction between the participants and reflecting different experiences in discussing the topics of interest [17]. Colleagues from the same PHCCs constituted a group, except one group comprising GP trainees who were interviewed at the local research and development (R&D) centre. All the other focus group interviews were carried through in PHCCs in conjunction with routinely scheduled meetings. In total, six focus groups with 2–8 participants were held. One of the authors (KBB), a clinically active GP and researcher, was moderator in all groups. KS, a GP, did not take part in the focus group interviews as she was lecturing in the education programme on sickness certification. ALÖ, a dentist and researcher with experience of qualitative methods, was supervisor. Observers (two different R&D secretaries) supported the moderator technically with recording, observed and took notes of the non-verbal communications between the participants [17]. The focus group interviews lasted between 50 and 90 min.

An interview guide was used to ensure that the areas of interest were covered [18]. The areas of topics to be discussed were *the role of the physician in the sickness certification process in collaboration with other parties, **the participants’ own experiences and, ***their views on regulations for sickness certification and rehabilitation. The discussion in each of the focus group interviews was introduced by the same opening question: ‘Sickness certification of patients – what does that mean to you?’ Thereafter, the discussion was left free and the moderator checked that the topics in the guide were covered and when needed, probing questions were posed. At the end of the focus group interviews the participants were given the opportunity to comment on topics they felt needed to be raised. The observer was also invited to ask additional questions based on the observations. After the interview when the participants had left the room, the moderator and the observer reflected on whether new topics had emerged and should be included in the guide. The recorded focus group interviews were transcribed verbatim by the observers. Each participant was assigned a code (the number of the focus group interview plus an individual code, e.g. FG 4:1) and information that could identify individuals or places was removed.

### Ethical considerations

An application for ethical vetting was submitted to the regional ethics committee in Gothenburg (570-14), which found that the project was not subject to the Swedish Ethical Review Act and had no objections to the study. All participants gave written consent to participate.

### Data analysis

Qualitative content analysis was deemed suitable for the analysis as the study had a mainly descriptive character but also with an aim of interpreting the underlying unspoken content [19]. Both manifest content (explicitly formulated in statements) and latent content (the underlying meaning of the text) were searched and interaction between the participants was identified. In a first step, the transcripts were read several times separately by all authors to get an overview. The second step was to identify meaning units i.e. expressions that highlighted topics and phenomena of interest throughout the transcripts. The meaning units were condensed, i.e. shortened to briefly describe the spoken statements. The third step was to sort these units into codes and thereafter into categories. The contents of the categories were ensured not to overlap. Finally, a theme presenting the latent content of the data was formulated. The authors had several meetings to discuss and reach consensus.

## Results

In total, 28 physicians participated in the focus group interviews. Most had received medical education in Sweden. Fifteen participants were specialists in general practice, five were GP trainees, four were interns and four were licensed physicians (Table 1). The age ranged from 30 to 60 years and 13 were women. The experience of PHC work ranged from a few months to 26 years. In the discussions, most of the topics of the interview guide were raised spontaneously and covered without any need of introduction by the moderator.

**Table 1.** Characteristics of 28 primary health care physicians participating in focus group interviews about sick leave.

Vocational status	GP	GP trainees	Interns	Licensed physicians
*N*	16	5	4	3
Women (of total)	9/16	1/5	4/4	0/3
Age range (years)	33–60	30–40	28–30	50–51
Time in general practice (years)	0.5–20	1–5	0	10–16
Education abroad	7	1	0	3
Private employment	6	2	1	0
Urban practice	2	1	1	0

GP: general practitioner.

**Figure 1.**
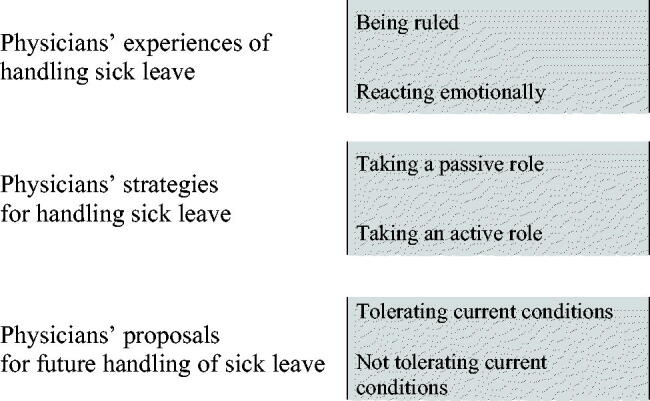
Three categories describing the content in physicians’ views of the sickness certification process.

**Figure 2.**
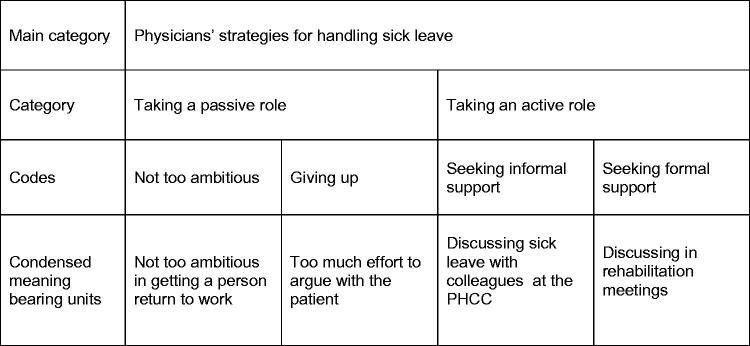
An example of the analysis from condensed meaning bearing units to the labelling of codes, categories and a main category. PHCC: primary health care centre.

The latent content of the focus group interviews was formulated in a theme: ‘The physicians perceived the sickness certification process as emotive and a challenge to master with differing demands and expectations from authorities, management and patients.’ Three main categories of the manifest content were identified, namely *the physicians’ experiences of handling sick leave; **the physicians’ strategies for handling sick leave; and ***the physicians’ proposals for future handling of sick leave (Figure 1). Within these three main categories further sub-categories were identified and labelled. Figure 2 shows an example of the analytic process from identifying meaning bearing units to the labelling of codes and categories.

### The physicians’ experiences of handling sick leave

This category was divided into two categories, the first one ‘being ruled’ was based on the participants’ narratives how their handling of sick leave was guided. The second category was labelled ‘reacting emotionally’ which includes the physicians’ personal emotions towards the certification task. This main category comprises the richest material in the study.

The category ‘being ruled’ included the participants’ reasoning that the patients’ needs always had to be related to laws and regulations in the field. The need of sick leave in patients with clear-cut diseases/diagnoses was regarded to generate few problems. However, in the case of more unclear medical conditions and uncharacteristic symptoms such as prolonged pain or mental illness, the sick leave decision and the rehabilitation process were complicated and prolonged. The complexity increased if social factors (failing family support, unemployment, unsatisfactory working conditions) influenced the patient’s ability to RTW. Sometimes these factors precluded the patients’ return to their former work and these circumstances ruled the decisions of sickness certification rather than the medical circumstances. These issues were discussed in all groups and were often spontaneously addressed as the first topic and generated intense interaction between the participants:

Psychiatric illnesses are most difficult – and longstanding cases – many components, not clean-cut (Focus group (FG) 3:5) – true (FG 3:3)

The mandatory certificate from the eighth day of sick leave was addressed in all groups. This was seen as leading to high pressure on the physicians as issuing of certificates was not accounted for in their mostly overbooked schedule. The laborious issuing of certificates and management of sick leave consumed time that the physicians instead wished they could have spent on medical care. The unlimited possibility of changing care provider could also mean that patients with ongoing sick leave wanted to change PHCC and unannounced turned up to get extension of an expiring sick leave period. This required immediate attention equivalent to emergency care. The consequence could be that patients with serious medical needs had to wait for care according to the participants:

In a way, it (sickness certification) is the heart attacks of primary care, nothing else has such high priority (FG 4:1)

The organization of health care was an issue raised in all groups and some level of conflict between different specialties could be discerned. Many participants described how other specialists transferred the issuing of sickness certificates for patients to PHC physicians without writing a formal referral:

When they are finished with the patient and … they can’t do anything further, then it is correct … but it lands in our lap (FG 2:2)

The decision support from the National Board of Health and Welfare was generally meant as a tutorial to facilitate certification and specifically helpful for physicians less fluent in Swedish. It could also be used as a pedagogical instrument in contact with patients. Also, ‘someone else’ could then be blamed as the authorities issued the rules of sick leave:

I usually say: I can issue a sickness certificate, but I don’t decide… it is up to you and the social insurance agency, ultimately they are the ones who decide (FG 6:1)

Likewise, the time limit for rehabilitation was discussed and seen as positive. It clarified that the insurance benefits were limited and put more pressure on all stakeholders involved:

As a matter of fact, I think it is better to have those time limits. You get regulations, it becomes less unclear…. it becomes more evident to people that sick leave is not a human right (FG 5:3)

The ICF manual from the WHO was however regarded being too laborious to use in the clinical situation. The participants even joked about the very comprehensive manual, ‘the little pamphlet’.

The social insurance officers (SIO) were spontaneously mentioned in all groups as important because they are the ones who make decisions about sick leave benefits. The SIOs lack medical education, which decreases their credibility according to the participants. Some SIOs were considered principled and bureaucratic, while others were pragmatic and prone to cooperate constructively. SIOs could be very demanding, not accepting certificates they considered incomplete, picking on details. On the other hand, SIOs were in some cases stationed at the PHCCs and readily available for advice, which was appreciated:

I have met many pragmatic, wise SIOs … (FG 1:2). They are very different … (FG 1:1) They must follow the same regulation … (FG 1:1) Of course they must…

Some think outside the box and others stay inside the box (FG 1:2)

The second subcategory of experiences contained the participants’ own emotions regarding issuing sickness certificates (‘reacting emotionally’), and these emotions might be described as ambivalent. The issuing was accepted by some participants, albeit not enjoyable. An emotional dilemma was when the patient’s wishes were opposite to the authorities’ rules for sick leave. The participants often felt powerless and left alone with the decisions. It was difficult to get ‘second opinions’ from other specialists, especially regarding patients with psychiatric diseases, which caused feelings of getting stuck with problems difficult to solve. Lack of training to assess the patient’s working conditions was common and often forced the participants to rely on the patient’s own history. Sometimes it was pure guesswork. A moral conflict between the tasks of exercising authority and giving care could be experienced if patients appealed to the physicians’ feelings, especially when sickness certification was not obvious. These encounters were challenging in prolonged sick leaves:

It is so sad, I feel so powerless, I have no one else … whenever I try to get a second opinion I get the cold shoulder … you are supposed to manage it by yourself. (FG 1:3)

If the patients are weeping pitifully, they do it to get our sympathy…. do you concede and say: I give you another week? … (FG 6:5) Well, we are also human beings … (FG 6:4)… Yes, yes of course we are (FG 6:5)

Lack of time and work overload could also elicit feelings of frustration. Especially when the reasons were out of control of the participants:

….my boss told me that I had to issue sickness certificates to three or four extra patients per day for a period …. I was so frustrated and angry. They backed off and apologised. (FG 4:2)

The participants felt scrutinized by the SIOs who criticized their inadequate ability to perform the necessary assessment of working incapability. The required amendments of certificates demanded extra work, often performed after office hours. Sometimes patients perceived that sick leave was declined by the SIOs because the physician produced inadequate certificates. A personal failure could be experienced:

It takes a long time to summarize the medical opinion … and records from different caregivers. The SIO does not accept it. You feel it personally … you are not good enough (FG 3:3)

### The physicians’ strategies for handling sick leave

This category was divided into two subcategories, describing two patterns of actions in handling sick leave: ‘taking a passive role’ and ‘taking an active role’. Both roles could be taken by an individual physician depending on the situation.

‘Taking an active role’ could be formally and/or informally done. Formal support could be collaboration meetings between the physician, the SIO, sometimes the employer and the patient. If the patient was unemployed, an official from the Swedish Public Employment Service also participated. Rehabilitation coordinators at the PHCCs could be a support, f especially if they kept contact with the patients and coordinated the activities in the sick leave process. Participants in all focus group interviews found the rehabilitation meetings very constructive with all stakeholders and facts at hand, discussing the patient’s ability to work. Participating in MMR teams also gave a broader knowledge of the patient’s abilities and hindrances for work.

It is outstanding that we sit together and reach consensus. Sometimes the patient needs (to hear) that you are distinct on what to do…… The patient can’t escape (FG 1:3)

Collegial meetings at the PHCCs were seen as a natural arena to discuss sick leave issues and to seek informal support. These were described as ‘debriefing sessions’ by some participants who used to participate in Balint groups [20]. The opportunity to discuss the care of the patients with other health professionals at the PHCC was also valuable. Informal support could involve joking about the difficulties and to get a good laughter together:

Yes, I have taken a half-day course in rehabilitation… now they say: that’s nice, now Dr A can take them all! … (FG 2:1) (-laughter) I was just going to propose that. (FG 2:2) I saw that in your eyes, oh yes, you were thrilled (FG 2:1)

With increasing experience that rehabilitation of patients often fails, the participants were more prone to become less active that is, taking on ‘a passive role’. The often high ambitions of a young physician could be replaced by thoughts like ‘you can’t help everyone’. Shielding oneself from the problems was a way to avoid disappointment and feelings of personal inadequacy, which helped the participants to survive professionally. The participants stated that they sometimes just gave up if the patient, relatives, employer or another caregiver required a certificate. It took too much effort to argue. If a physician was part of an MMR team, where there were conflicting opinions about patients’ sick-leave, it was difficult to argue against the team. The participants felt they only executed decisions of others:

I am not guilty so now I do what I can – not too ambitious to get this person back to work …. And I try not to blame myself (FG 5:2)

The team rather obstructed the return to work (FG 2:2)… You felt like a sickness certification monkey … My only task was to issue a sickness certification (FG 2:1)

### The physicians’ proposals for future handling of sick leave

Two clear ways of actions could be discerned: ‘tolerating current conditions’ and ‘not tolerating current conditions’.

Some participants *tolerated the current conditions* and accepted the current sickness certification process as being part of their job. Some had positive experiences, within the prevailing system, of cases earlier deemed hopeless still, the patient got back to work. This rendered great satisfaction, worth all the effort and also boosted the self-esteem and incentives to help patients RTW. Compassion for the patients and awareness of the poor socio-economic conditions for many patients was also important for job satisfaction:

All my education is paid if I prevent some young persons from getting early disability pensions … Yes absolutely (FG 5:3)

‘Not tolerating current conditions’ included suggestions for future work with sick leave and rehabilitation which was spontaneously discussed. Other staff such as nurses, vocational therapists or physiotherapists might be entrusted the administration of shorter periods of sick leave. Participants with experience of social security systems from other countries proposed, for instance, that PHC physicians, after investigation for diagnosis and initiating treatment, referred the patient to specialized rehabilitation physicians or teams:

A special sick-listing physician! Can it become a reality? If so, it would be so wonderful! (FG 2:2)… Yes, it would be a relief … Yes, the work would be much more enjoyable, easier (FG 2:3). It would make family medicine more attractive (FG 2:1)

A more radical solution for the individual physician when not tolerating the working conditions was opting out of the speciality. This was brought up spontaneously in all focus group interviews. The heavy task of sickness certification and the associated process was not what they had expected when they chose the specialty. They felt poorly trained to assess the working ability associated with the diseases or illnesses of the patients. More experienced participants feared that it would be increasingly difficult to recruit young physicians to the specialty.

This was not what I thought when I signed up. Sickness certification is burdensome and makes the work as a GP heavy (FG 1:5)

## Discussion

The qualitative data analysed in this study revealed that the participating physicians perceived that their work task with sickness certification was emotive and a challenge to master. The process contained differing demands and expectations from authorities, management and patients. The physicians’ coping strategies for the task included both active measures and passive adaptation to the circumstances. The main suggestion for the future was that physicians should be relieved of the heavy administrative task in order to instead devote themselves to the medical care of patients.

### Strengths and limitations

Colleagues from the same PHCC in the focus group interviews reflected the current staffing with a mix of age and professional experience of participants to explore the research questions from various aspects. This is a strength as earlier studies in the field have most often only included experienced GPs [11,13,14]. A potential drawback was that the discussion might be hampered if senior physicians dominated the discussion and younger less experienced ones suppressed their views [21]. The moderator was aware of this and encouraged more silent colleagues to express their views. On the other hand, meeting with your peers at the PHCC could have made the participants more comfortable. Participation in the focus group interviews was voluntary and physicians not wanting to express their views would probably not join in.

The trustworthiness of the results was evaluated in terms of credibility, dependability and transferability [19]. Credibility is the ability to focus on the intended aim. To fulfil this criterion, we chose participants with various background to address the questions from different angles and focus group interviews to increase the variation in data and to study the interaction between participants. Predefined topics in the interview guide could increase the risk of bias in the focus group interviews, therefore the guide was thoroughly discussed from different perspectives by the authors. It turned out that nearly all topics were spontaneously discussed and very few new topics had to be added to the guide during the study. In the analysis, topics discussed with interaction between the participants were considered of more importance. With regard to the research question, the data collection was carried out over a limited time period to ensure that circumstances were consistent for the focus group interviews. Thus, the dependability criterion was met. The transferability of the findings to other settings and groups relies on the choice and description of the context and the presentation of the results. Thus, we put much effort into choosing participants from different settings in PHC and presenting both the most commonly and alternatively expressed experiences and feelings. Thus, the qualitative information gained in the study can be seen as relevant for similar contexts.

Organizational issues, including legislation, cooperation with authorities and practical formalities emerged as the most engaging issue in the focus group interviews and took up most of the time. Some of the new regulations such as the decision support, and rehabilitation coordinators were perceived as a helpful support in the sick leave process, similar to the findings in an earlier Swedish study [14]. The work could also be relieved by the stricter assessments by the SIOs and the absolute time limit of 2.5 years for the rehabilitation. Thus, the physician’s guilt feelings could be transferred to other parties [22]. In line with this, it was found in a study from Norway that the strict conditions in Norwegian legislation provided a relief for the physicians [23]. The social insurance officers played a crucial role during sickness certification both positive in cooperation and presence at the health care centre and negative in demanding amendments in the certificates. In a Swedish interview study GPs described unsanctioned techniques in order to get the sick leave approved [24], for instance exaggerations of problems in the best interest of the patient. This was not found in our study but all parties in the sickness certification process adjusted their acting according to the laws and regulations, not only physicians but also the social security officials, employers as well as the patients.

However, the way the sickness certification process is organized had more negative than positive consequences according to the participants in the study. The extensive paperwork was regarded exhausting. Patients needing renewal of sickness certificates might suddenly appear at the PHCC without referral, and an already tight work schedule could be overloaded. The free choice of caregiver in Swedish PHC [8] meant that new patients with ongoing sick leave from other PHCCs could turn up demanding immediate consultations, according to the participants. In a survey, Norwegian GPs expressed concern that patients not getting their requested sickness certification would turn to another GP and thus might jeopardize the business [25]. This might occur in a system where there is competition for patients however, no such concerns were expressed in the current study.

The work situation for the physicians in our study appeared to be most demanding. The dual role as helper on the one side and representative of authorities on the other in sickness certification was seen as problematic, confirming findings of an earlier focus group interview study with Swedish GPs and GP trainees [14]. Moreover, the feeling of loneliness and lack of support in the work with sickness certification and rehabilitation was frustrating. A disturbing finding was that the problematic work environment brought a notion of resignation. Many had seriously considered leaving the specialty and the burden of sickness certification contributed to this. This is in line with a large survey showing that 52% of Swedish PHC physicians regarded sickness certification as burdensome and a vocational environmental problem [13]. It is therefore necessary to reduce the potential for conflicts between physicians, patients and other stakeholders, to clarify their respective roles in the process and to increase the access to rehabilitation specialists as described in the earlier literature review [1]. Further, such measures might make the specialty of general practice a more attractive choice to young physicians.

The physicians in our study used different, more or less conscious, strategies to cope with the situation. These were both active strategies, for instance participating in Balint groups [20] or using the decision support, and passive strategies such as being less engaged or indulgent. These strategies could be expressed and used by the same physician depending on the context. Experienced physicians seemed to have a more pragmatic attitude which might be natural, as they are probably more secure in their professional role. They tried their best in cases of prolonged sick leave and avoided taking their responsibility too seriously.

The help of other professionals for instance in assessing the patient’s work situation was positively perceived and has likewise been found a relief in a recent Swedish study [14]. The participants appreciated local rehabilitation coordinators being active in contacts with patients, evaluating treatment and encouraging rehabilitation. However, rehabilitation coordinators were not available in all PHCCs and the personal skills of the coordinator varied. Also, the MMR teams could be problematic according to our findings as there could be disagreement about the patient’s situation within the team. The benefit and contribution of MMR teams on RTW have been addressed in a review with 14 studies, mostly of musculoskeletal disorders but also of mental health [26]. The review revealed that the time to RTW, cumulative sickness absence, the proportion of RTW at short-term follow-up or ever did not differ from care as usual. In a more recent randomized controlled study a multidisciplinary intervention did not facilitate RTW or decrease health care utilization compared to ordinary case management and could even reduce the chance of RTW in some patients [27].

The problems concerning sickness certification has also been described from other countries, especially in Scandinavia and northern Europe. Results similar to ours were described in a Nordic survey [25] and in qualitative studies from Ireland [28] and Finland [29]. These studies describe the same kind of problems with conflicts with other stakeholders, lack of time and insufficient knowledge on how to assess the patients’ work ability. Since the interviews took place the sick leave situation in Sweden has become even tougher which underpins the results of this study and calls for further action.

For the future, it was spontaneously suggested in all groups but one that patients after a defined sick leave period should be transferred to specialized rehabilitation teams. This idea was usually raised by participants with experience of education or work abroad. Apart from lifting a burden from the physicians’ shoulders such specialized teams might contribute to a higher degree of quality and equity in assessments of work ability which was one of the aims of the reform in 2005. According to a Swedish survey [30] occupational health physicians have work situations that are more favourable compared to GPs regarding sickness certifications. Further, they have high professional competence for assessment of patients’ work capacity and organizational support for their work. More extensive use of rehabilitation coordinators and increased contacts with social security officers might also help the physicians in their work with sickness certification and contribute to make the PHC an attractive workplace for physicians.

### Meaning of the study

Political decisions on laws and regulations for sickness certification impacted the primary health care making the physicians’ work difficult and burdensome. Their views and suggestions should be carefully considered in future organization of primary care.
